# Screening differential circular RNAs expression profiles in Vulvar Lichen Sclerosus

**DOI:** 10.1186/s12938-022-01013-7

**Published:** 2022-08-01

**Authors:** Min Yang, Kailv Sun, Jianmin Chang

**Affiliations:** Department of Dermatology, Beijing Hospital, National Center of Gerontology, Institute of Geriatric Medicine, Chinese Academy of Medical Sciences, Beijing, 100730 China

**Keywords:** Vulvar lichen sclerosus, circRNAs, mRNAs, lncRNAs

## Abstract

**Background:**

Vulvar lichen sclerosus (VLS) is one of the most common clinical manifestations of vulva. Thirteen percent of women have symptomatic vulvar diseases. The aim of this study is to investigate the expression profile of circular RNA (circRNAs) in vulvar lichen sclerosus, and to identify the underlying core genes of VLS.

**Methods:**

We removed rRNA for sequencing, and screened the differentially expressed messenger RNA (mRNAs), long non-coding RNA (lncRNAs) and single-stranded circRNA in 20 groups of VLS tissues and 20 groups of healthy female vulvar skin tissues. Bioinformatics analysis was used to analyze its potential functions.

**Results:**

A total of 2545 differentially expressed mRNAs were assessed in VLS patients, of which 1541 samples were up-regulated and 1004 samples were down-regulated. A total of 1453 differentially expressed lncRNAs were assessed, of which 812 samples were up-regulated and 641 samples were down-regulated. A total of 79 differentially expressed circRNAs were assessed, of which 54 were up-regulated and 25 were down-regulated. The differential expression of circRNAs was closely related to biological processes and molecular functions. The differences in circRNAs were mainly related to the “human T-cell leukemia virus 1 infection” signaling pathway and the “axon guidance” signaling pathway.

**Conclusion:**

The profile of abnormal regulation of circRNA exists in VLS. According to biological informatics analysis, the dysregulation of circRNAs may be related to the pathogenesis and pathological process of VLS.

**Supplementary Information:**

The online version contains supplementary material available at 10.1186/s12938-022-01013-7.

## Background

VLS is one of the most common clinical manifestations of vulva. Thirteen percent of women have symptomatic vulvar diseases [1]. International Society for the Study of Vulvovaginal Diseases (ISSVD) classification in 1975, vague terms are used for vulva lichen sclerosis, such as leukoplakia, nodules, and vulva dystrophy [2]. The current classification of ISSVD includes the disease entity with exogenegative skin disease, which is non-neoplastic and non-infectious in nature [3]. However, they have the potential to transform into vulvar intraepithelial neoplasia (VIN) and keratinized vulvar carcinoma [4]. VLS is a chronic inflammation whose pathogenesis is still unknown. However, studies had speculated that there were three pathophysiological mechanisms of VLS: infectious disease etiology, primary immune disorders and isotraumatopic reactions [5]. Clinicopathological studies of VLS indicated that the progression of VLS was related to the density of inflammatory infiltration and the number of pathogens [6].

There are many treatments available for VLS, such as local steroid therapy, intra-lesional steroid therapy, topical calcineurin inhibitors and so on [7]. Nevertheless, there are relatively few studies on VLS gene expression profile. Therefore, it is an urgent task to identify molecular biomarkers and explore the potential mechanisms of VLS, which may contribute to the development of novel diagnostic and therapeutic approaches for VLS [8, 9]. In recent years, bioinformatics methods have been widely used to analyze microarray data in order to identify differentially expressed genes (DEGs) and perform various analyses [10]. In our study, two groups of samples were sequenced, and VLS-related circRNAs were screened and predicted by the Gene Ontology (GO) enrichment and the Kyoto Encyclopedia of Genes and Genomes (KEGG) enrichment.

As early as 20 years ago, a new type of RNA was identified; instead of a linear structure, this special kind of RNA is covalently closed and was named circular RNA (circRNA). CircRNAs have no free 5′ cap structure and 3′ poly (A) structure, and are insensitive to nucleases, so they are more stable than linear RNA [11]. CircRNAs are mainly localized in the cytoplasm and can be stored in exosomes [12, 13]. Subsequently, endogenous circRNAs had been identified in human ETS-1 gene and mouse SRY gene, but only a small number of circRNAs had been identified at that time [14, 15]. Moreover, it was regarded as a by-product of abnormal shear without regulatory potential [16]. With the deepening of research, circRNAs are not splicing by-products, they are widely derived, conservative, stable, and tissue-specific, and play a variety of functional roles in the growth and development of organisms [17–19]. In recent years, studies have found that circRNAs derived from human INK4A/ARF and CDR1 genes have an influence on human atherosclerosis and are involved in the regulation of mRNA expression, provided a new dawn for circRNA research [20]. With the rapid development of high-throughput sequencing and bioinformatics analysis, tens of thousands of circRNAs have been identified in cells and tissues of different species, most of which are conserved and stable across different species, and their expression is cell, tissue and developmental stage specific [21, 22]. Although a large number of circRNAs have been identified, the research on the function of circRNAs and the mechanism of circRNAs formation have only just begun. The types of circRNAs can be classified into the following four types according to their sources: (1) full exon-type circRNAs; (2) EicircRNAs in intron–exon combinations; (3) lassotype ciRNA composed of introns; (4) circRNAs produced by cyclization of viral RNA genomes, tRNAs, rRNAs, snRNAs [12, 23–25]. The understanding of circRNAs is getting deeper and deeper, and its biological functions in various physiological and pathological states have gradually became a new research hotspot. In summary, the main known functions of circRNAs include transcriptional regulation [26], miRNA adsorption [27], binding with functional proteins to regulate cell function [28] and protein translation [29], and insertion into the genome after reverse transcription to become pseudogenes [30], etc. To date, little research have been done on the relationship between circRNAs and VLS [31]. The correlation between the significant changes in the expression of circRNAs and the pathogenicity of VLS has not been clearly studied. The purpose of this study was to mine the differential expression of circRNAs in VLS and determine the potential role of selected genes in the circRNAs etiology of VLS. In addition, this article conducted further research on the potential impact of circRNAs on VLS.

## Results

### mRNAs expression profile of VLS patients mRNAs

**Fig. 1:**
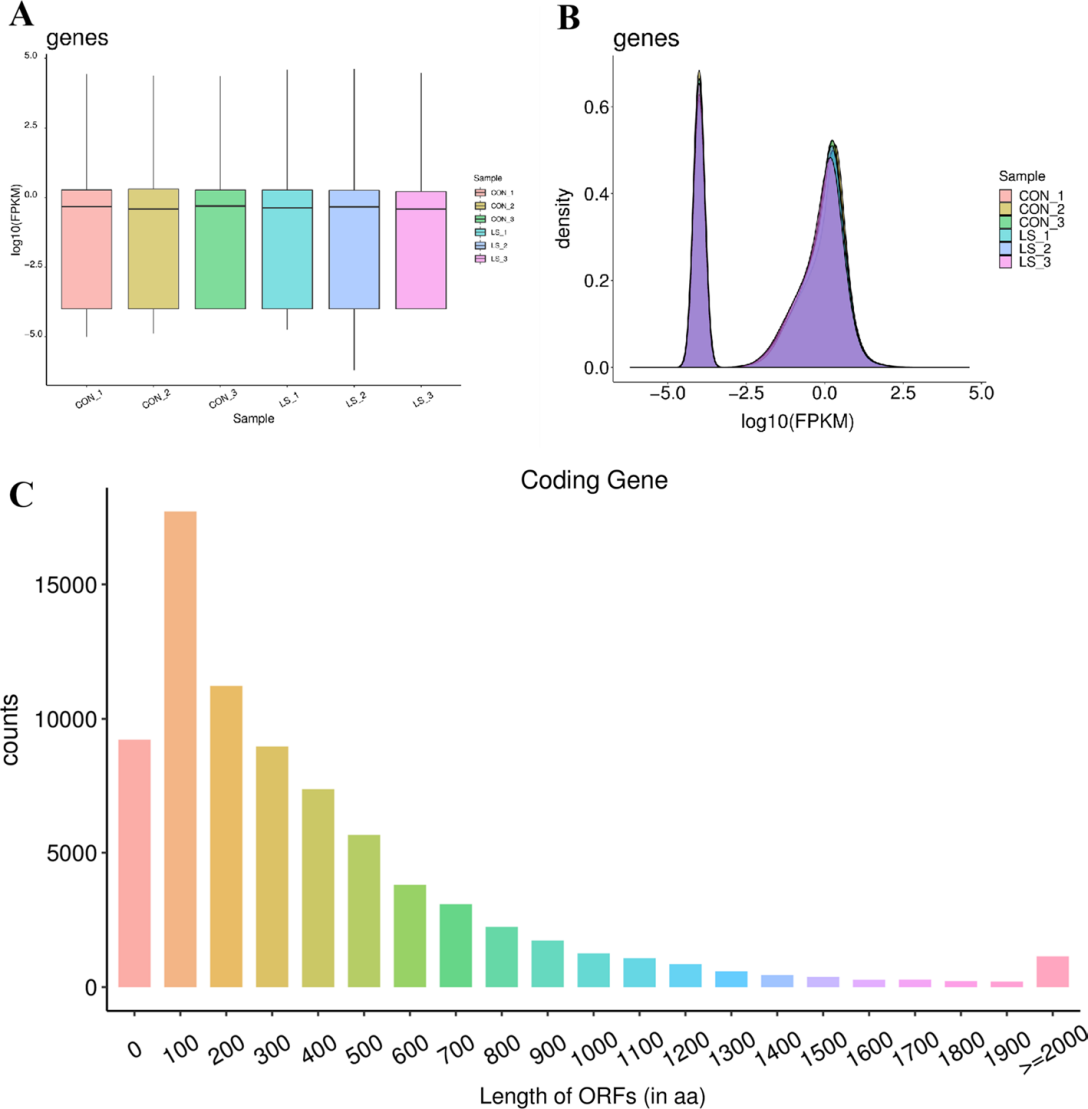
mRNAs expression profile in VLS tissues. **A** VLS samples of gene expression value distribution statistics. The abscissa was the sample name, the ordinate was log10 (FPKM), and the box chart for each region corresponded to five statistics (top to bottom for maximum, upper quartile, median, lower quartile, and minimum). **B** mRNAs expression density distribution. In the ideal state, the expression density map of each sample should conform to the normal distribution, and the expression trend of biological duplicate samples should be consistent. The abscissa was log10 (FPKM) and the ordinate was the gene density. **C** Distribution of mRNA ORF length. The horizontal axis showed the length of the mRNA ORF, and the y axis showed the quantity of mRNAs was equal to the ORF length

The circRNAs upstream of the target gene can participate in the mediation of circRNA gene mRNAs. Therefore, we explored the abnormally expressed mRNAs in VLS. Sequencing was performed in three samples of vulva lichen scleroid tissue and three samples of normal tissue. Through the analysis, 262,922 transcripts were identified, corresponding to 73,150 genes. FPKM (Fragments Per Kilobase of exon model Per Million mapped reads) was used to measure the abundance of gene expression, and the expression abundance of known genes in different samples was counted by FPKM. FPKM denoted the number of sequence fragments per thousand transcriptome per million sequence bases, and FPKM value denoted the gene expression level. The results are shown in Table 1. The expression values in the above table were distributed statistically, and the expression levels of VLS genes were understood from the overall level by using the FPKM box chart of the samples, and the reproducibility of the designed samples was preliminarily judged (Fig. 1A). We standardized the test results of different samples and compared the trend of mRNAs expression among different samples. The difference expression of mRNAs was displayed with density distribution diagram (Fig. 1B). According to the length distribution of the ORF region of mRNA, the statistical histogram was performed (Fig. 1C).

**Table 1:** Statistical table of gene expression value distribution of VLS samples

Sample	Exp gene	Min	1st Qu	Median	Mean	3rd Qu	Max	Sd	Sum
con_1	73150	0.00	0.00	0.47	3.15	1.87	27,468.06	167.51	230,237.02
con_2	73150	0.00	0.00	0.39	2.87	2.02	23,597.42	125.30	209,870.47
con_3	73150	0.00	0.00	0.50	2.90	1.86	22,750.92	135.94	212,251.61
Ls_1	73150	0.00	0.00	0.43	3.48	1.86	39,044.21	187.73	254,289.76
Ls_2	73150	0.00	0.00	0.46	3.39	1.81	40,945.13	187.91	246,122.50
Ls_3	73150	0.00	0.00	0.38	3.23	1.63	30,278.75	155.99	236,486.17

### The mRNAs were differentially expressed in VLS patients

**Fig. 2:**
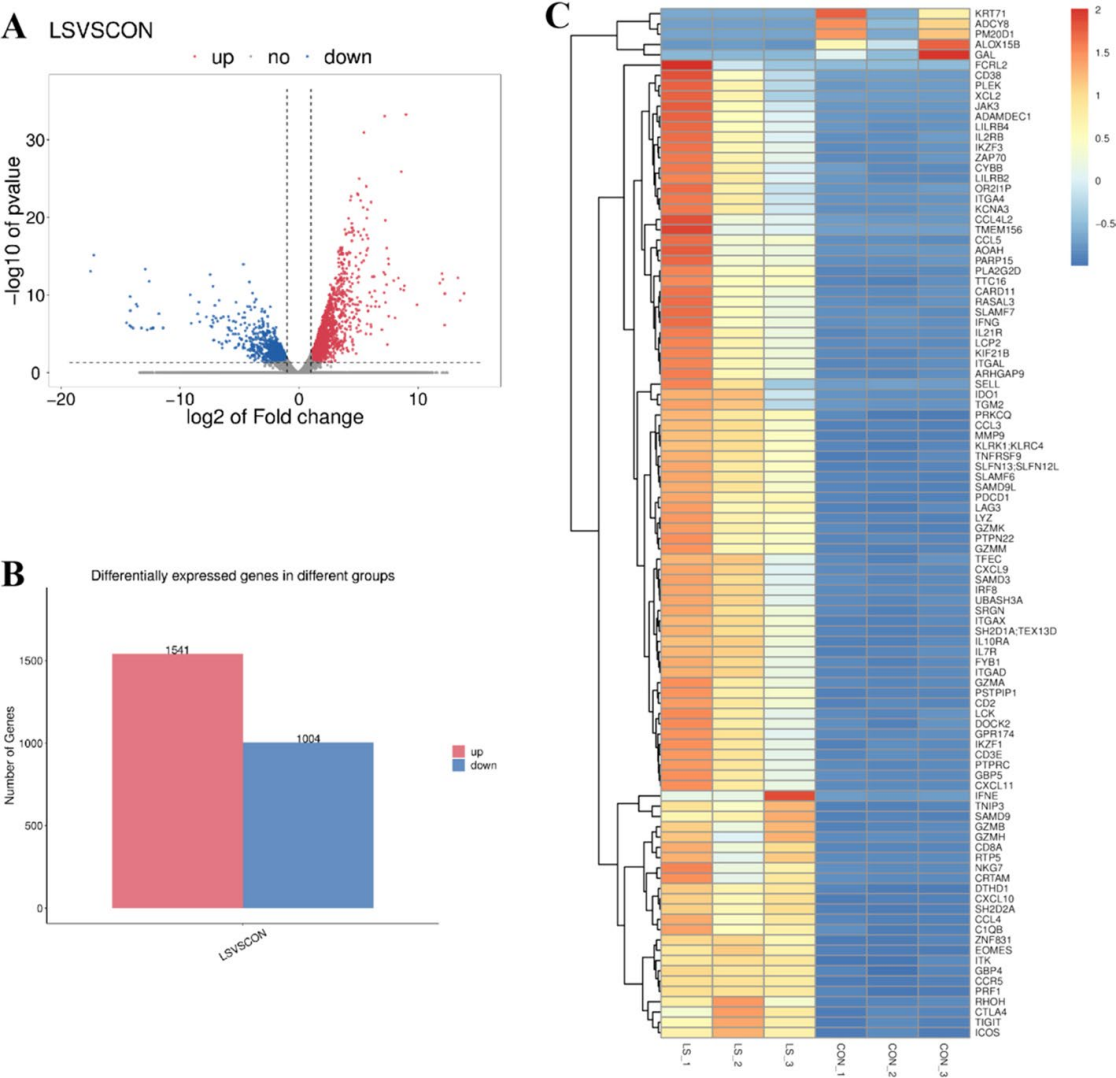
VLS tissues and normal tissues directly differentially expressed mRNA. **A** Volcanic map of VLS differential gene expression level. The *x* axis represented the normalized difference (VLS group/normal group). The *y* axis represented the normalized *P* value. In the figure, red represented the up-regulated significantly differentially expressed genes, blue represented the down-regulated significantly differentially expressed genes, and the gray dots represented the non-significantly differentially expressed genes. **B** Statistical chart of down-regulated frequency of VLS genes with significantly different expression. The red column represented up-regulated gene frequency, and the blue column represented down-regulated gene frequency. **C** Cluster analysis diagram of VLS differential gene expression level. The horizontal axis was the sample (VLS group/normal group), and the vertical axis is the gene. Different colors represent different gene expression levels, and the color ranges from blue to white to red to indicate the expression amount (log10(FPKM + 1)) from low to high. High expression genes were in red and low expression genes were in dark blue

Based on screening threshold, the figure in the volcano identified a total of 2545 differentially expressed mRNA, logFC | > 1, *P* values < 0.05, of which 1541 were raised and 1004 lowered (Fig. 2A). Table 2 lists the ten mRNAs with the largest expression differences (up-regulated and down-regulated). Figure 2B shows a statistical histogram based on the down-regulation frequency of significantly differentially expressed genes. Cluster analysis was performed in the VLS chain-specific library. In order to better intuitively reflect the clustering expression pattern, we used log10 (FPKM + 1) to process the gene expression (Fig. 2C).

**Table 2:** Differences in mRNA between LS and control groups

Gene_name	Up/down	Log2(fc)	*p*-val
PLA2G2D	Up	8.99	0.00
CXCL10	Up	7.18	0.00
MMP9	Up	5.45	0.00
CXCL9	Up	8.59	0.00
SLAMF6	Up	5.06	0.00
ADCY8	Down	− 12.91	0.00
GAL	Down	− 17.52	0.00
PM20D1	Down	− 7.47	0.00
UGT2A1	Down	− 12.57	0.00
DCT	Down	− 4.17	0.00

### Pathway enrichment analysis of differentially expressed genes

**Fig. 3:**
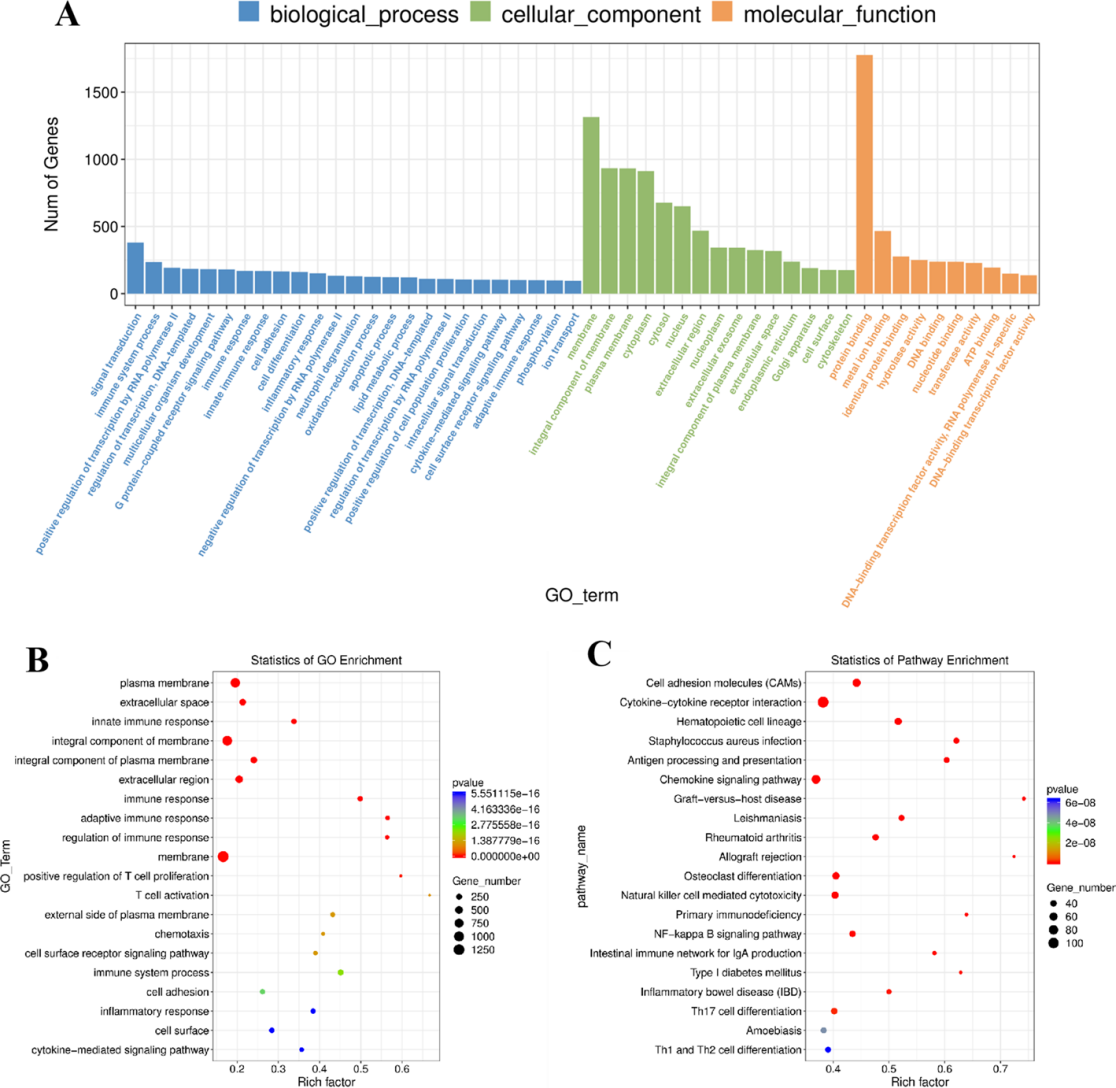
Pathway enrichment analysis of differentially expressed genes. **A** GO enrichment analysis of VLS differential genes. The functional characterization message of the calculation was the amount of abnormal genes enriched into the entry. **B** GO enrichment scatter plot of VLS differential genes. In the horizontal axis, Rich Factor represents the number of differential genes located in the GO/the total number of genes located in the GO (Rich Factor = S gene number/B gene number). The higher the Rich Factor was the higher the enrichment degree of GO. The ordinate was GO_TERM which was the GO function comment. In the scatter plot, the size of the dot represented the number of genes with significant differences that S gene number matches to a single GO, the color of the dot represented the P value of enrichment analysis, namely the significance of enrichment, and the *p*-value less than 0.05 indicated significant enrichment. **C** KEGG enrichment analysis of VLS differential genes. The value of − log10 (*p* value) was important for enrichment. The larger value, the more significant the consequence. The amount of genes was equal to the amount of genes enriched in the clause. The richness factors were the percentage of the amount of genes in the way, which was abnormally expressed in the overall amount of genes in the way. The greater enrichment factors, the higher enrichment degrees. Vertical axis was the appellation of enrichment project

The GO function enrichment analysis and KEGG pathway enrichment analysis of mRNA host genes showed significant difference (*P* < 0.05). A total of 902 GO BPs (biological processes), 109GO CC (cellular components), 235 GO MF (molecular functions), 1246 functional and KEGG pathways were enriched. Differentially expressed mRNAs were involved in signal transduction and protein binding in the plasma membrane (Fig. 3A). The GO enrichment analysis results were statistically analyzed by GGplot2, and the results were presented in the form of scatter plots (Fig. 3B). ‘Cytokine–cytokine receptor interactions’ and ‘chemokine signaling pathways’ are significant pathways (Fig. 3C).

### Expression profile of lncRNAs in VLS patients

**Fig. 4:**
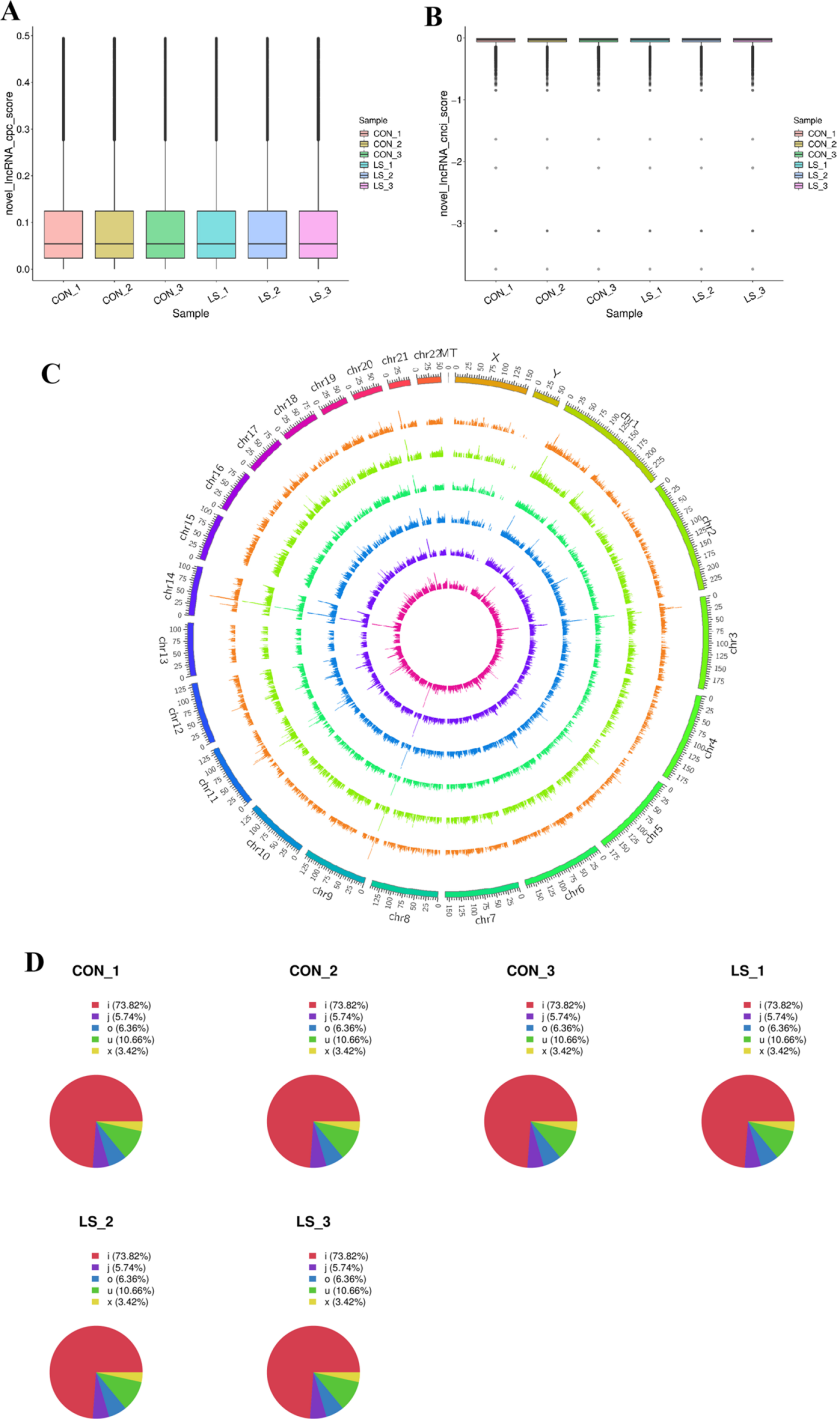
mRNA expression profile in VLS tissues. **A** Prediction analysis of lncRNA CPC in VLS samples. The abscissa was the sample name and the ordinate was log10(FPKM). **B** Prediction analysis of lncRNA CNCI in VLS samples. The abscissa was the sample name and the ordinate was log10(FPKM). **C** Visualization results of lncRNAs genomes from different samples. Genome mapping was carried out according to different classification of lncRNAs, and every 25 MB of each chromosome was taken as the basic unit, and the expression level of lncRNAs in each segment of the lncRNAs genome in different samples was counted and mapped during the visualization mapping. The larger inner green ring represented all lncRNAs detected. Smaller inner loops indicated differentially expressed lncRNA folding changes > 2 and *P* value < 0.05. The up–down regulation of lncRNA has been marked with red and blue bars. **D** Pie chart of different class code ratios of lncRNA

CircRNAs upstream of target genes can participate in the mediation of LncRNAs of circRNA genes. Therefore, we explored the abnormally expressed LncRNAs in VLS. We removed known mRNAs and transcripts less than 200 bp, and then used CPC (Coding Potential Calculator) and CNCI (Coding non-coding Index) software to predict the remaining transcripts by lncRNAs. If these remaining transcripts had the potential to encode proteins, we classified them as NOVEL mRNAs and then defined them as mRNA and filtered them. We performed a statistical analysis of the CPC and CNCI scores of lncRNAs in each sample, and the results are shown in box diagrams (Fig. 4A, B). To facilitate observation, we visualized the genome of lncRNAs in different samples (Fig. 4C). We made statistics on the percentages of different class codes of lncRNAs in each sample, and the results are shown in a pie chart (Fig. 4D).

### Differential expression of lncRNAs in VLS patients

**Fig. 5:**
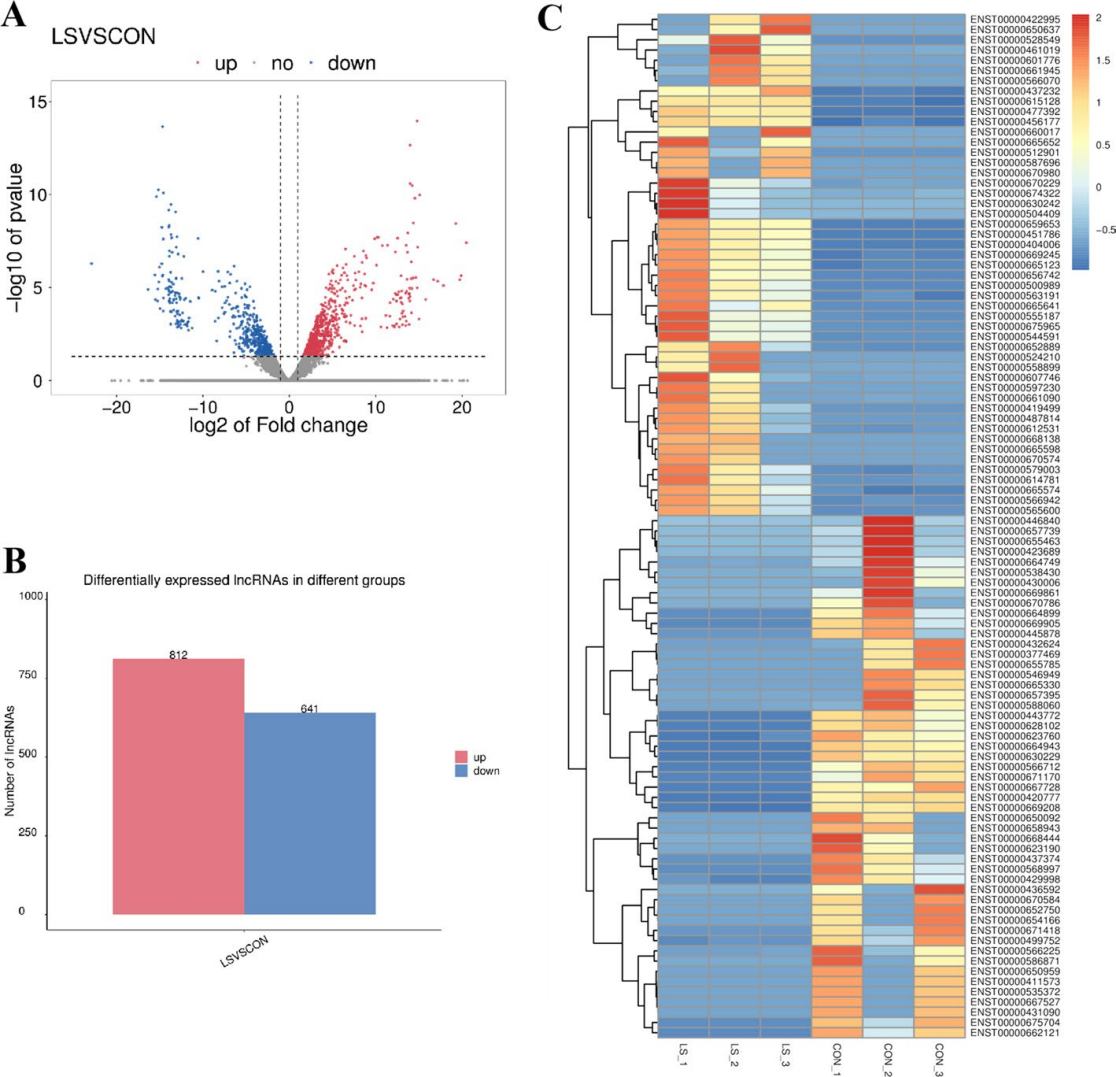
Direct differentially expressed lncRNAs in VLS tissues and normal tissues. **A** Volcanic map of VLS differential gene expression level. The *x* axis represented the normalized difference (VLS group/normal group), and the *y* axis represented the normalized *P* value. In figure, red represented the up-regulated significantly differentially expressed genes, blue represented the down-regulated significantly differentially expressed genes, and the gray dots represented the non-significantly differentially expressed genes. **B** Statistical chart of down-regulated frequency of VLS genes with significantly different expression. The red column represented up-regulated gene frequency, and the blue column represented down-regulated gene frequency. **C** Cluster analysis diagram of VLS differential gene expression level. The horizontal axis was the sample (VLS group/normal group), and the vertical axis was the gene. Different colors represented different gene expression levels, and the color ranged from blue to white to red to indicate the expression amount (log10(FPKM + 1)) from low to high. High expression genes were in red and low expression genes were in dark blue

Based on screening threshold, the figure in the volcano identified a total of 1453 differentially expressed LncRNAs, logFC | > 1, *P* values < 0.05, of which 812 were raised, lowered 641 (Fig. 5A). Table 3 lists ten lncRNAs with the largest expression differences (up-regulated and down-regulated). Figure 5B shows a statistical histogram based on the down-regulation frequency of significantly differentially expressed genes. Cluster analysis was performed in the VLS chain-specific library. In order to better intuitively reflect the clustering expression pattern, we used log10 (FPKM + 1) to process the gene expression (Fig. 5C).

**Table 3:** LncRNA differences between LS and control groups

Gene_name	Up/down	Log2(fc)	*p*-val
MSC-AS1	Up	13.92	0.00
TFAP2A-AS1	Down	− 15.37	0.00
LINC01409	Down	− 13.96	0.00
TBX5-AS1	Up	12.53	0.00
AC015911	Up	9.88	0.00
RNF216P1	Up	10.77	0.00
LINC00885	Down	− 12.2	0.00
LINC00861	Up	5.04	0.00
LINC01698	Down	− 9.99	0.00
LINC00402	Up	8.02	0.00

### circRNA expression profile in VLS patients

**Fig. 6:**
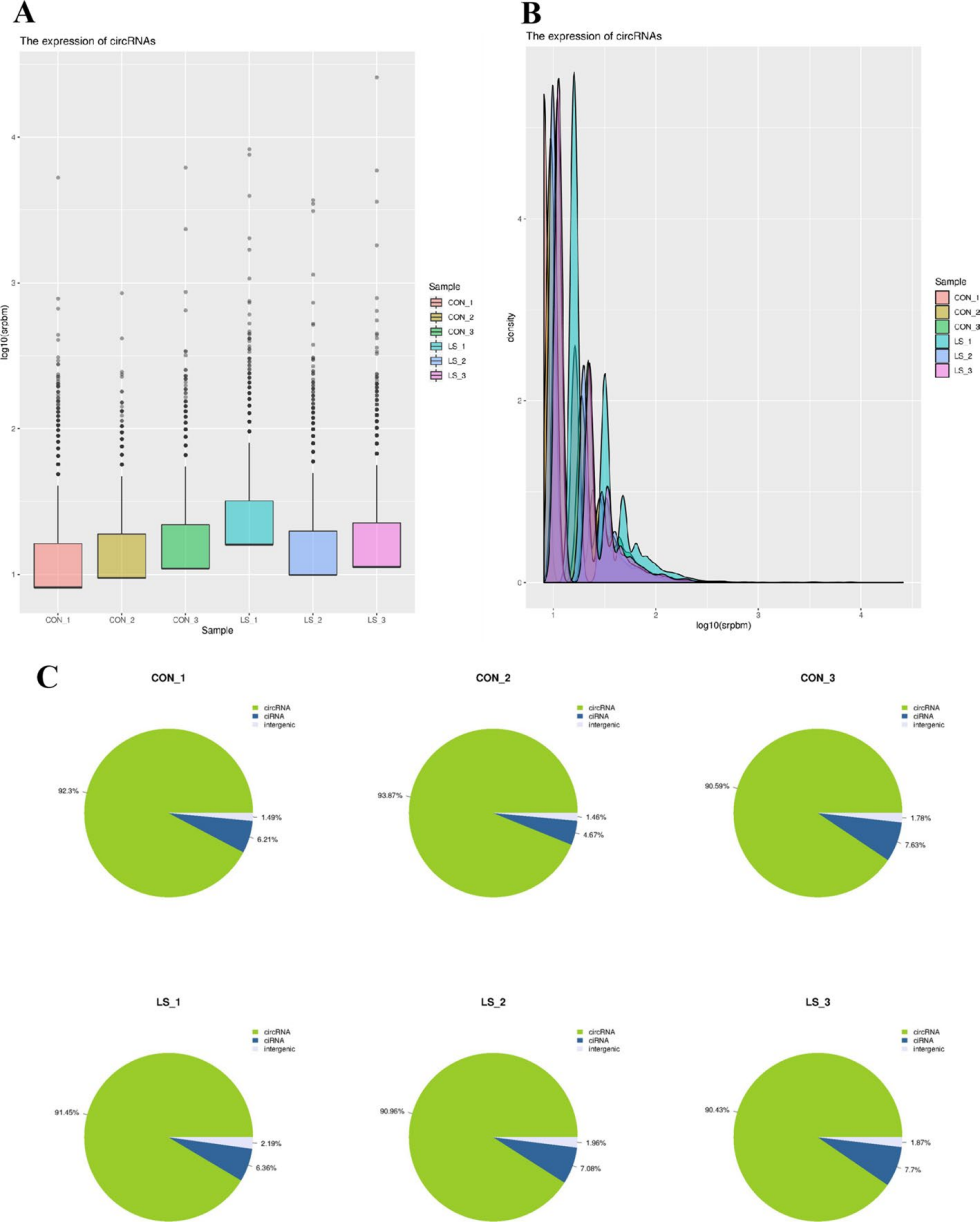
Expression profile of lncRNA in VLS tissues. **A** Statistical distribution of gene expression values in VLS samples. The abscissa was the sample name, the ordinate was log10(FPKM), and the box chart for each region corresponds to five statistics (top to bottom for maximum, upper quartile, median, lower quartile, and minimum). **B** Expression density distribution of lncRNAs. The abscissa was log10(FPKM) and the ordinate was the gene density. **C** Distribution of lncRNAs species. ① Full exon-type circRNAs; ② EicircRNAs in intron–exon combinations; ③ lassotype circRNAs composed of introns; ④ CircRNAs generated by viral RNA genomes, tRNAs, rRNAs, snRNAs and other circRNAs

The FPKM box chart of the samples was used to understand the gene expression level of VLS from the overall level and initially judged the repeatability of the designed samples (Fig. 6A). The respective expression levels of different samples were processed to compare the changes in the expression trend of circRNAs among different samples. We had analyzed the expression distribution in circRNAs, and the results were displayed with a density distribution diagram (Fig. 6B). The pie chart analysis was performed according to the number distribution of circRNAs types (Fig. 6C).

### circRNAs were differentially expressed in VLS patients

**Fig. 7:**
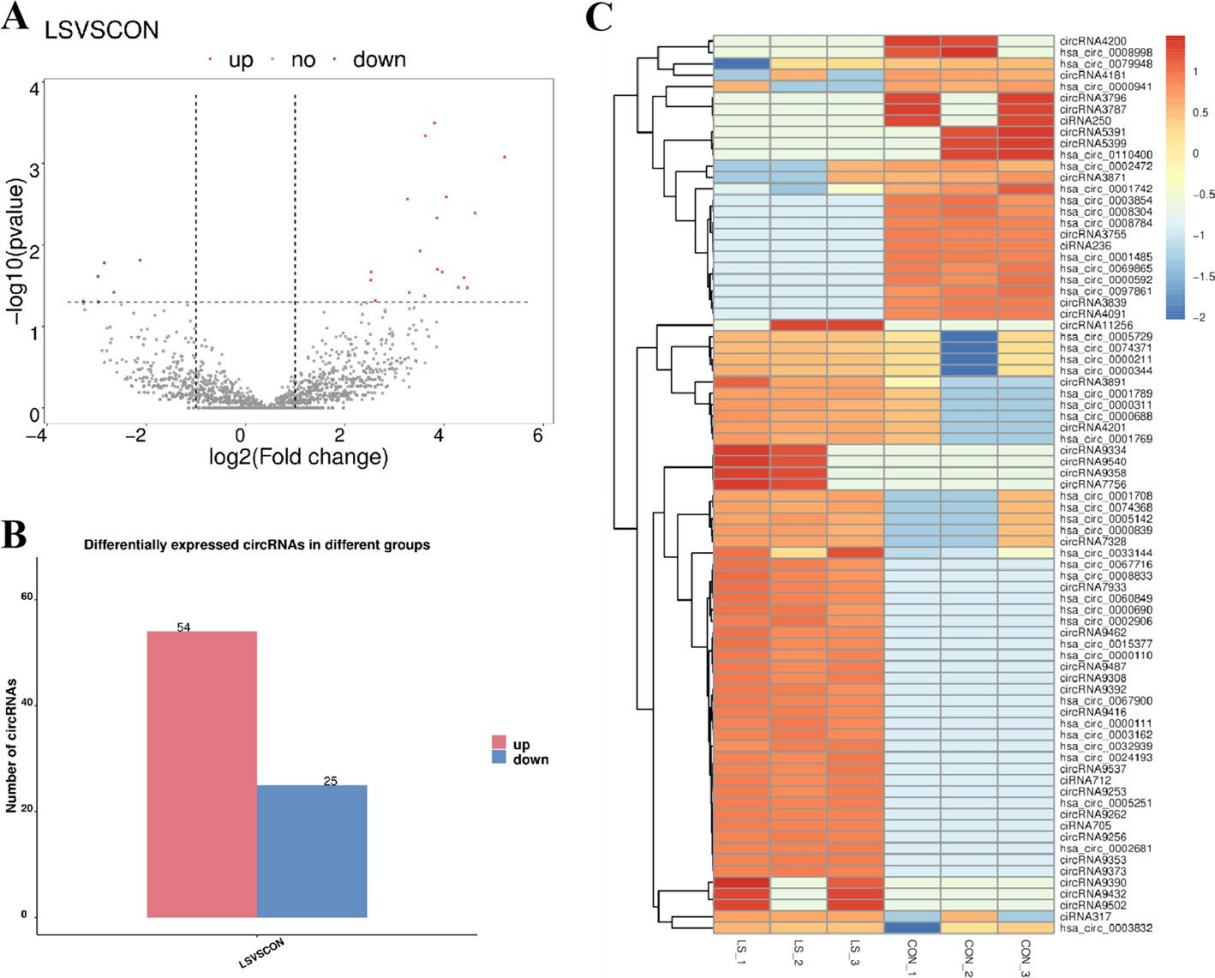
circRNAs were directly differentially expressed in VLS tissues and normal tissues. **A** Volcanic map of VLS differential gene expression level. The horizontal axis represented the normalized difference (VLS group/normal group), and the vertical axis represented the normalized *P* value. In the figure, red represented the up-regulated significantly differentially expressed genes, blue represented the down-regulated significantly differentially expressed genes, and the gray dots represented the non-significantly differentially expressed genes. **B** Statistical chart of down-regulated frequency of VLS genes with significantly different expression. The red column represented up-regulated gene frequency, and the blue column represented down-regulated gene frequency. **C** Cluster analysis diagram of VLS differential gene expression level. The horizontal axis was the sample (VLS group/normal group), and the vertical axis was the gene. Different colors represented different gene expression levels, and the color ranged from blue to white to red to indicate the expression amount (log10(FPKM + 1)) from low to high. High expression genes were in red and low expression genes were in dark blue

Based on screening threshold, the figure in the volcano identified a total of 79 differentially expressed circRNAs, logFC | > 1, *P* values < 0.05, of which 54 were raised, lowered 25 (Fig. 7A). The 10 circRNAs are summarized with the most significant expression differences (5 up-regulated and 5 down-regulated) in Table 4. Figure 7B shows a statistical histogram based on the down-regulation frequency of significantly differentially expressed genes. Cluster analysis was performed in the VLS chain-specific library. In order to better intuitively reflect the clustering expression pattern, we used log10 (FPKM + 1) to process the gene expression (Fig. 7C).

**Table 4:** circRNA difference between LS and control groups

Gene_name	Up/down	Log2(fc)	*p*-val
has_circ_0000211	Up	3.62	0.00
has_circ_0001789	Up	5.22	0.00
has_circ_0005729	Up	3.26	0.00
has_circ_0000839	Up	4.62	0.00
has_circ_0000344	Up	3.51	0.00
has_circ_0002472	Down	− 3.27	0.00
has_circ_0008059	Down	− 3.26	0.00
has_circ_0004440	Down	− 3.24	0.00
has_circ_0009021	Down	− 2.25	0.00
has_circ_0001742	Down	− 2.12	0.00

### Pathway enrichment analysis of differentially expressed genes

**Fig. 8:**
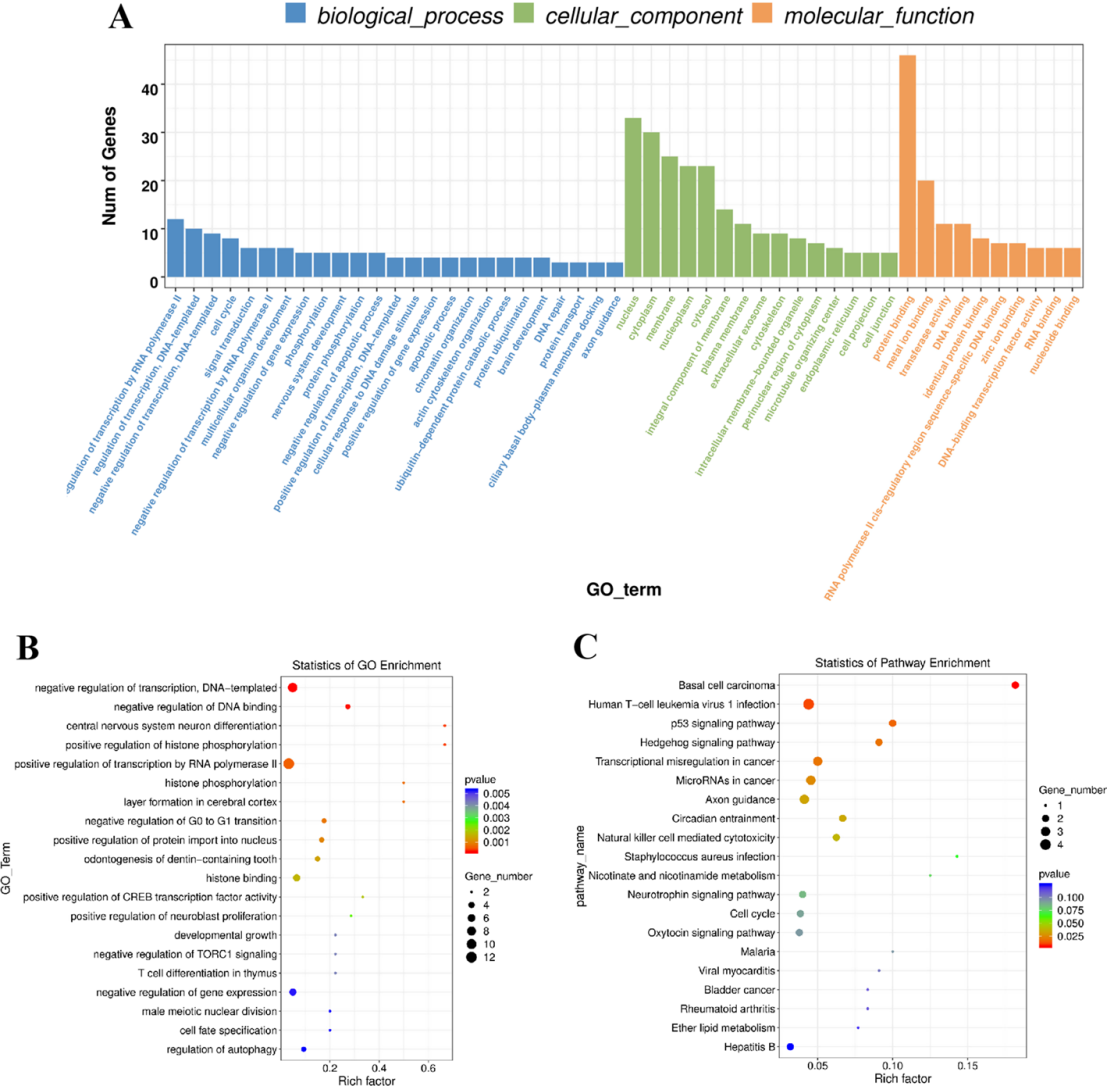
Pathway enrichment analysis of differentially expressed genes. **A** GO enrichment analysis of VLS differential genes. The functional characterization information of the calculation was the amount of abnormal genes enriched into the item. **B** GO enrichment scatter plot of VLS differential genes. In the horizontal axis, Rich Factor represented the number of differential genes located in the GO/the total number of genes located in the GO (Rich Factor = S gene number/B gene number). The higher the Rich Factor was, the higher the enrichment degree of GO. The ordinate was GO_TERM which was the GO function comment. In the scatter plot, the size of the dot represented the number of genes with significant differences that S gene number matched to a single GO, the color of the dot represented the *P* value of enrichment analysis, namely the significance of enrichment, and the *p*-value less than 0.05 indicates significant enrichment. **C** KEGG enrichment analysis of VLS differential genes. The value of − log10 (*p* value) was important for enrichment. The larger value, the more significant the consequence. The amount of genes was equal to the amount of genes enriched in the clause. The richness factors were the percentage of the amount of genes in the way, which was abnormally expressed in the overall amount of genes in the way. The greater enrichment factors, the higher enrichment degrees. Vertical axis was the appellation of enrichment project

The enrichment analysis between GO function and KEGG pathway of circRNAs host genes showed significant differences (*P* < 0.05). After research, we found that the differentially expressed circRNAs in VLS enriched a total of 229 GO-BPs, 22 GO-CCs and 22 GO-MFs. In addition, we also found that 273 functions were related to the KEGG pathway. The differently expression of circRNAs were associated with negative regulation of DNA transcription and positive regulation of RNA polymerase II transcription in the nucleus (Fig. 8A). The GO enrichment analysis results were statistically analyzed by GGplot2, and the results were presented in the form of scatter plots (Fig. 8B). The ‘human T-cell leukemia virus 1 infection’ and ‘axon-directed’ signaling pathways are significant pathways (Fig. 8C).

### Interactions between circRNAs and miRNAs were analyzed

The interaction of circRNAs–miRNAs were predicted by TargetScan and miRANDA. TargetScan predicted miRNAs target based on seed region. MiRanda was mainly based on the binding free energy of circRNAs and miRNAs. The smaller the free energy, the stronger the binding ability of the two. The results are shown in Table 5.

**Table 5:** circRNA combination of microRNAs

circRNA	miRNA	miranda_Energy
has_circ_0000110	has-miR-19a-5p	− 15.2
has_circ_0000211	has-miR-224-5p	− 22.85
has_circ_0000311	has-miR-215-3p	− 20.13
has_circ_0000344	has-miR-196a-3p	− 16.56
has_circ_0000592	has-miR-181a-2-3p	− 13.54
has_circ_0000690	has-miR-198	− 20.39
has_circ_0000839	has-miR-221-5p	− 18.07
has_circ_0000941	has-miR-29a-3p	− 16.74
has_circ_0001485	has-miR-182-3p	− 13.06
has_circ_0001742	has-miR-133a-5p	− 17.74

## Discussion

It has been proved that circRNA plays a significant role in the occurrence and progression of disease. In addition, circRNAs have become a hotspot for transcriptome and RNA research. According to the idiographic structure and complicated functional mechanisms of circRNAs, the researches of circRNAs have only come into the field of view in recent years [32]. Recent studies have shown that circRNA is closely related to the occurrence and development of a variety of diseases and tumors [33–35]. To clarify the potential role of circRNAs in VLS, our experiment verified the differential expressions of circRNAs in VLS patient tissues and performed functional analysis. We randomly sampled vulvar skin tissues from 20 patients and 20 healthy women. All patients were untreated patients and their VLS was in the acute phase. All patients were untreated patients and their VLS was in the acute phase. As this study mainly studied VS, the study patients were all female patients and no male patients. All patients in the study underwent a biopsy, and samples were taken after the biopsy.

In this study, we observed that 79 circRNAs were differentially expressed in VLS tissues, of which 54 circRNAs expressions were significantly increased and 25 circRNAs expressions were significantly reduced. This study listed 5 significantly up-regulated (hsa_circ_0000211, hsa_circ_0001789, hsa_circ_0005729, hsa_circ_0000839, hsa_circ_0000344) and down-regulated circRNAs (hsa_circ_0002472, hsa_circ_0008059, hsa_circ_0004440, hsa_circ_0109021, hsa_circ_0001742). These circRNAs may be related to the pathogenesis and pathological process of VLS.

We found through volcano graph analysis that the expression of circRNAs in the figure was similar to the expression of circRNAs in the VLS sick person group. Through cluster analysis, we found that there were obvious discrepancies in circRNAs expressions between vulvar skin of healthy women and skin of VLS patients. We found that compared with healthy female vulvar skin, the expression of some circRNAs in the skin of VLS patients had significant differences, which indicated that there were significant differences in the expression of certain circulating RNAs between the vulva skin tissues of healthy women and the tissues of patients with VLS. Whether these differential circRNAs can affect the treatment of VLS still needs further research.

The enrichment analysis between KEGG pathway and GO indicated that the unusual expressions of circRNAs participated in multiple biology processes and signal-transduction pathways. Biological information analysis of abnormal genes found that differentially expressed circRNAs were related to the negative regulation of DNA transcription and the positive regulation of RNA polymerase II transcription in the nucleus. KEGG pathway analysis showed that the “human T-cell leukemia virus type 1 infection” signaling pathway and the “axon guidance” signaling pathway were significantly enriched with differential circRNAs. This suggested that the “human T-cell leukemia virus type 1 infection” signaling pathway and the “axon guidance” signaling pathway may be related to the occurrence and development of VLS.

An increasing number of studies have shown that circRNAs play crucial parts in many biological functions, for instance, circRNAs act as ceRNAs or miRNAs sponges, which regulate gene expression by competitively binding miRNAs with MRE (microRNA-responsive element, MRE). In recent years, studies have revealed that circRNAs upstream of target genes participate in the mediation of miRNAs, mRNAs and ceRNAs of circRNA genes. Therefore, circRNAs affect the occurrences and pathological processes of a variety of diseases. For example, circ-cdr1as can act as a sponge for hsa-miR-7 and modulate person epidermal GF receptors by competitively binding to miR-7 [20, 36]. In addition, circRNAs can specifically bind to many kinds of latent factors, for example splicing factors muscle (MBL/MBNL1) [37]. Our research showed that the following 10 pairs of circRNAs may interact with miRNAs: hsa circ_0000110 and hsa-miR-19a-5p, hsa circ 0000211 and hsa-miR-224-5p, hsa_circ_0000311 and hsa-miR-215-3p, hsa_circ_0000344 and hsa-miR-196a-3p, hsa_circ_0000592 and hsa-miR-181a-2-3p, hsa_circ_0000690 and hsa-miR-198, hsa_circ_0000839 and hsa-miR-221-5p, hsa_circ_0000941 and hsa-miR-29a-3p, hsa_circ_0001485 and hsa-miR-182-3p, hsa_circ_0001742 and hsa-miR-133a-5p. These interacting circRNAs and miRNAs may be related to the molecular mechanism of VLS pathogenicity.

## Conclusion

In conclusion, this study has identified the differential expression of circRNA in VLS (hsa_circ_0000211, hsa_circ_0001789, hsa_circ_0005729, hsa_circ_0000839, hsa_circ_000034, hsa_circ_0002472, hsa_circ_0008059, hsa_circ_0004440, hsa_circ_0109021, hsa_circ_0001742). Bioinformatics analysis shows that circRNAs-related diseases may be related to VLS, and the signal pathway of ‘human T-cell leukemia virus type 1 infection’ and the signal pathway of “axon guidance” may also affect the pathological process of VLS. This study reveals a total of ten pairs of circRNAs and miRNAs interacting. How these interacting circRNAs–miRNAs affect the pathological findings of VLS by regulating downstream mRNAs remains to be studied. This study could expand the perspective of VLS gene research and build the basis in future research on the part of circRNAs in VLS.

## Materials and methods

### Clinical specimen acquisition

Specimens of LS tissue and vulva skin tissue of healthy women were obtained from 20 LS patients and 20 healthy women treated in Beijing Hospital. The research was divided into experimental group and control group with age matching. This study was approved by the Ethics Committee of Beijing Hospital and a written informed consent was obtained from patients. (For details of 20 LS patients and 20 healthy women, please see Additional file 1: Table S1.)

### Preparation of sequencing library and circRNA sequencing

Among the 20 specimens randomly extracted from the three LS tissues, total RNA was extracted from this sample, and the chain-specific library was constructed by the method of rRNAs depletion. After the library passed the quality inspection, Illumina Novaseq™ 6000 was used for sequencing, with a double-ended reading length of 2 * 150 bp (PE150) [24]. Raw data generated by sequencing need to be preprocessed, cutadpter was used to filter unqualified sequences to get clean data, and then the next step was analyzed. The specific processing steps were as follows: (1) removed reads with adaptor; (2) removal of the proportion of reads containing more than 5% N (N means that the base information cannot be determined); (3) removed low-quality reads (the number of bases with mass value *Q* ≤ 10 accounted for more than 20% of the whole read); (4) the original sequencing quantity, effective sequencing quantity, Q20, Q30 and GC content were counted and comprehensively evaluated.

### Cluster analysis of differential gene expression levels

Differential gene clustering analysis was used to determine the clustering patterns of regulatory patterns of genes under different experimental conditions. Cluster analysis of genes was conducted according to the similarity of gene expression profiles of samples to intuitively display the expression of genes in different samples (or different treatments), thus obtaining biology-related information. In order to better intuitively reflect the clustering expression pattern, we used log10 (FPKM + 1) for gene expression display. The horizontal axis was the sample and the vertical axis was the gene. Different colors represented different gene expression levels, and the color ranged from blue to white to red to indicate the expression level (log10(FPKM + 1)) from low to high. High expression genes were in red and low expression genes are in dark blue.

### Enrichment analysis of differential genes

#### GO functional significance enrichment analysis

Gene Ontology (GO) is an international standardized system of gene function classification. It provided a dynamically updated controlled vocabulary to comprehensively describe the properties of genes and Gene produced in an organism. There were three ontologies in GO, which described molecular function, cellular component and biological process of gene, respectively. The basic unit of GO was term (term, node), and each term corresponded to an attribute. The GO functional significance enrichment analysis first mapped all the differentially expressed genes to each term in the Gene Ontology database, calculated the number of genes in each term, and then applied hypergeometric test to find the GO items significantly enriched in differentially expressed genes compared with the entire genome background.

#### KEGG enrichment analysis

In organisms, different genes coordinated their biological functions with each other, and Pathway-based analysis was helpful for further understanding the biological functions of genes. KEGG was the main public database on Pathways. Pathway significance enrichment analysis used KEGG Pathway as the unit, and hypergeometric tests were used to identify the pathways that were significantly enriched in differentially expressed genes compared with the overall genome background.

### Statistical analysis

We use SPSS 22.0 software for statistical analysis of the data. Categorical variables are expressed as numbers or percentages, and continuous variables are expressed as mean ± standard deviation. Categorical variables were analyzed by Fisher’s exact test, and continuous variables were analyzed by between-group *t* test. *T* test was used to compare whether there were significant differences in the expression of RNA in VLS. Spearman was used to analyze the correlation of RNA in VLS. *p* < 0.05 and *p* < 0.01 were used to establish statistically significant differences. “*” means statistical difference (*P* < 0.05), “**” means statistical difference (*P* < 0.01).

## Supplementary Information

**Additional file 1.** Immune-related genes and apoptosis-related genes.

## Data Availability

The datasets used or analyzed during the current study are available from the corresponding author on reasonable request.

## Abbreviations

mRNA: Messenger RNA
lncRNAs: Long non-coding RNA
circRNAs: Single-stranded circular RNA
VLS: Vulvar lichen sclerosus
ISSVD: International Society for the Study of Vulvovaginal Diseases
VIN: Vulvar intraepithelial neoplasia
DEG: Differentially expressed genes
GO: Gene Ontology
KEGG: Kyoto Encyclopedia of Genes and Genomes
